# Has the biobank bubble burst? Withstanding the challenges for sustainable biobanking in the digital era

**DOI:** 10.1186/s12910-016-0124-2

**Published:** 2016-07-12

**Authors:** Don Chalmers, Dianne Nicol, Jane Kaye, Jessica Bell, Alastair V. Campbell, Calvin W. L. Ho, Kazuto Kato, Jusaku Minari, Chih-hsing Ho, Colin Mitchell, Fruzsina Molnár-Gábor, Margaret Otlowski, Daniel Thiel, Stephanie M. Fullerton, Tess Whitton

**Affiliations:** Centre for Law and Genetics, Faculty of Law, University of Tasmania, Hobart, Tasmania Australia; Centre for Health, Law and Emerging Technologies (HeLEX), Nuffield Department of Population Health, University of Oxford, Oxford, UK; Centre for Biomedical Ethics, Yong Loo Lin School of Medicine, National University of Singapore, Singapore, Singapore; Department of Biomedical Ethics and Public Policy, Graduate School of Medicine, Osaka University, Osaka, Japan; Heidelberg Academy of Sciences and Humanities, Heidelberg, Germany; Department of Health, Management and Policy, School of Public Health, University of Michigan, Ann Arbor, Michigan USA; Department of Bioethics and Humanities, University of Washington, Seattle, WA USA; Academia Sinica, Taipei, Taiwan

**Keywords:** Biobanks, Sustainable biobanking, Comparative review, Medical research ethics, Genetics and genomics, Personalised medicine, Precision medicine

## Abstract

Biobanks have been heralded as essential tools for translating biomedical research into practice, driving precision medicine to improve pathways for global healthcare treatment and services. Many nations have established specific governance systems to facilitate research and to address the complex ethical, legal and social challenges that they present, but this has not lead to uniformity across the world. Despite significant progress in responding to the ethical, legal and social implications of biobanking, operational, sustainability and funding challenges continue to emerge. No coherent strategy has yet been identified for addressing them. This has brought into question the overall viability and usefulness of biobanks in light of the significant resources required to keep them running. This review sets out the challenges that the biobanking community has had to overcome since their inception in the early 2000s. The first section provides a brief outline of the diversity in biobank and regulatory architecture in seven countries: Australia, Germany, Japan, Singapore, Taiwan, the UK, and the USA. The article then discusses four waves of responses to biobanking challenges. This article had its genesis in a discussion on biobanks during the Centre for Health, Law and Emerging Technologies (HeLEX) conference in Oxford UK, co-sponsored by the Centre for Law and Genetics (University of Tasmania). This article aims to provide a review of the issues associated with biobank practices and governance, with a view to informing the future course of both large-scale and smaller scale biobanks.

## Background

Over the past 20 years, there has been considerable investment in biobanking and research infrastructure in scientifically- advanced countries because of the perceived research benefits they provide. Biobanks are variable in size and purpose, and may comprise single user, disease-specific tissue and data collections or multi-user, population banks, or anything in between [1]. Meir et al. broadly define biobanks as: ‘collection[s] of biological samples and associated data, systematically organized for use by stakeholders, such as researchers and health care providers’ [2]. This definition is expansive enough to include conventional tissue banks and other collections, such as bloodspots from newborn screening programs. Although some mention is made of these collections later in this article, it is not the intention of the article to address the particular issues associated with these types of collections. Rather, this article focuses primarily on biobanks that are established and maintained specifically for use by multiple researchers.

In essence, these research-focused biobanks comprise collections of human tissue linked with genetic, genealogical, health and other personal information, which can be used for a number of research purposes and from which a multitude of different datasets can be extracted [3, 4]. They are seen to accelerate the research effort because researchers do not have to expend valuable time and funds on the collection, storage and curation of human tissue samples and data. The research power of biobank datasets is considerably enhanced if they can be combined with equivalent datasets from other biobanks, but the provisos are that there must be uniformity in the ways that they are collected, extracted and coded and that ethical, legal and social implications (ELSI) are appropriately taken into account. The greatest value comes from these rich resources if they can be combined together in the form of ‘Big Data’ [5] for use in addressing research questions of global significance.

The early 21st century saw a significant upsurge in public and private investment in the establishment of new research-focused biobanks. According to a BCC market research report published in 2011, the global biobanking market was $141 billion in 2010, and was projected to increase by 30 % between 2010 and 2015, to an estimated $183 billion [6]. Investment has been focused on the establishment of population biobanks and cohort studies as well as the organisation of existing smaller collections under broader research infrastructure. This coincides with considerable investment in sequencing technology and the funding of research consortia and disease networks with specific research objectives. However, this funding commitment has not been sustained in all countries, as Australia and Singapore have seen withdrawal of funding from biobanks.

Public funds have also been invested in small–scale biobanks, which may be disease or project specific, as well organ banks and repositories of samples derived from clinical pathology [1]. More recently, public funding has been invested in the establishment of international collaborative consortia, to enhance the research power of biobank data sets that may be considerably strengthened if they are combined with equivalent data sets from other biobanks and data repositories.

This article starts with a review of biobank initiatives in some of the most research-active countries globally, together with a brief overview of the key aspects of their regulatory frameworks. This sets the scene for a broader review of the key challenges for biobanking over the past twenty years. This review is demarcated by three separate ‘waves’. The first wave saw particular focus on the local and global governance frameworks that needed to be put in place to ensure that the particular ELSI of biobanking were appropriately addressed. In the second wave, the focus was directed towards standardisation and harmonisation to facilitate combination and sharing of biobank datasets. The third wave of challenges focuses on the financial sustainability of biobanking. Funding for biobank maintenance has always been a challenge [1], but it has come into sharp focus recently as funders question their commitment to providing support in perpetuity. This article then looks prospectively to a future ‘fourth wave’, where we propose that technological developments and new strategies for community engagement could coalesce and, where complex infrastructures and financial burdens are minimised without compromising the research value of biobanking or public trust in the biobanking enterprise.

## The global environment for biobanking: investment, infrastructure and governance

The brief country-by-country review presented in this part is intended to illustrate the variability of approaches to biobanking and biobank governance. In parallel with investment and the international priority of building infrastructure for research, new governance structures for biobanking have emerged. Some countries have introduced biobank-specific legislation while others have included provisions in existing law or delegated the responsibility to national bodies to develop guidance on biobanking. In addition, many of the bigger and more recent biobanks have developed internal ethics and governance oversight frameworks, including guidelines and policies [7].

## Australia

Since the 2000s, Australia has seen the implementation of a number of mostly disease specific biobanking facilities. The premier national funding agency, the National Health and Medical Research Council (NHMRC) largely supported these biobanks, with a focus on cancer. Samples and clinical data in breast, prostate, ovarian, leukaemia, lymphoma and other blood cancers were collected and stored, involving some very large disease cohorts, as well as rare and more general cancers. In alignment with the international movements on good governance, the NHMRC included a chapter on databanks in the 2007 edition of the *National Statement on Ethical Conduct in Human Research* [8] and also issued an *Information Paper on Biobanks* in 2010 [9], which provided guidance on many issues relating specifically to biobanking procedures. In 2011, the Commonwealth Department of Industry, Innovation, Science and Research (DIISR) issued a *Strategic Roadmap for Australian Research Infrastructure* for sector review [10]. This Roadmap acknowledged the key role of biobanking in health research and also in underpinning population health research platforms. In 2013 the national budget to fund biobanking was cut, significantly affecting the landscape for biobanking in Australia.

## Germany

Germany saw a rise in the number of biobanks for specific disease-related research as well as diagnostic and research needs of healthcare institutions in the early 2000s, with federal government support for biobanking continuing. Since 2011, five centralised biobanks (cBMBs) within the ‘National Initiative for Biomaterial Banks’ have been funded by the German Federal Ministry for Education and Research (BMBF), recently extending the promotion to a sixth biobank alliance. In 2013, the BMBF set up the German Biobank Node (GBN). The BMBF has also promoted six German Centres of Health Research (DZG) equipped with biobanks within its Health Research framework program. These are inter-centre initiatives for combatting widespread diseases. Since 2014, the ‘National Cohort’, a cross-financed large-scale study involving 200,000 participants for over ten years at 18 research centres, has been brought under the aegis of the GBN, which thus functions as the central contact point and networking agent for the biobank community [11].

The German Ethics Council (known as the National Ethics Council until 2007) published two extensive opinions on biobanking in 2004 and 2010 [12, 13]. In the second opinion, the Council made a detailed five-pillar legislative proposal for the regulation of biobanks, aimed at enhancing protection of donors’ interests, including at the international level and recommended that statutory provisions on human biobanks for research be passed [13]. Following extensive discussions in the German Bundestag in 2012, the proposed legislation, based on the opinion of the Ethics Council, was rejected. The main reasons for this rejection included lack of necessity, absence of comparable legislation in other countries and the perceived need to avoid bureaucratisation of research [14]. This outcome was much to the relief of German biobanks and research centres that feared the existence of smaller biobanks would be compromised if the legislation was passed, and that its adoption (and forced secrecy) would hinder international cooperation [15, 16]. More recently, in 2015, a group of university lawyers drafted alternative draft legislation on biobanks [17].

## Japan

In Japan, several large-scale biobank projects have started since the 2000s. One of the representative projects is Biobank Japan, started in 2003 and samples collected from 200,000 patients [18]. The current focus of Biobank Japan is to create a third phase of 38 common diseases involving 100,000 patients [19]. Several diseases, such as dementia and depression have been newly added as target diseases. Another national scale project, the National Center Biobank Network (NCBN) started in 2011 [20], to integrate their activities and to accelerate the efficient use of the collected samples. This project is run by the six National Centers for advanced and specialized medical care in Japan. Currently over 150,000 samples are attached to the network, organised in a single database called “NCBN Electronic-Catalogue-based Database” [21]. The latest project regarding large-scale biobanking is the Tohoku Medical Megabank Organization (ToMMo) [22] launched in 2012, as a large-scale cohort study to promotes biobank activities and to collect samples from 150,000 participants. By late 2015, ToMMo has completed a few thousand whole genome sequences (WGS), and resulting data have been used to construct a reference panel of 1,070 Japanese individuals [23].

In April in 2015, a new funding agency, the Japan Agency for Medical Research and Development (AMED) [24], was created for biomedical research and development. The AMED has been revisiting its policy and project managements regarding genome research and its medical application. The interim report on the genomic medicine by the Headquarters for Healthcare Policy in the Japanese government [25] in July 2015, will serve as a key guiding document for the AMED as well as other related Ministries. This interim report states that while several large biobanks have been established successfully, they need to be proactively utilised for the implementation of clinical medicine. Strong emphasis is placed on the term “genomic medicine” and its implementation. This could be interpreted as indicative of pressure on biobanks to demonstrate actual benefits to medicine and society.

## Singapore

In 2002, the Singapore Tissue Network (STN) was established with public funding, and subsequently reconstituted as the Singapore Bio-bank (SBB), which was intended to function as the national not-for-profit tissue bank to facilitate translational and population-based epidemiological research [26]. Apart from the SBB, other sizeable tissue banks in Singapore include the SingHealth Tissue Repository (STR) [27] and the National University Health System Tissue Repository (NUHS TR) [28]. Unlike the STR and the NUHS TR, which collect mainly residual biological samples from patients, the SBB’s collection was derived from researchers and, to a more limited extent, from the STR and NUHS TR. However, the SBB was discontinued in September 2011 due to concerns over long-term operational viability and financial sustainability, triggered by its low utilisation rate [26, 29].

While a national-level biobank might not have been evaluated to be a sustainable undertaking, a variety of bio-specimen collections and practices have subsisted and thrived in Singapore. This may have, in part, prompted the enactment of the *Human Biomedical Research Act* (HBRA) on 18 August 2015, which establishes a relatively comprehensive legal framework on research involving human participants (other than clinical trials) and their biological materials. This legislation essentially builds on the system of ethical guidelines instituted by the government, on the advice and recommendations of its Bioethics Advisory Committee (BAC) report on ‘Human Tissue Research’ in November 2002 [30]. These guidelines apply to all locally registered medical practitioners and publicly funded research [31]. The ethical principles embodied in the guidelines include the primacy of the welfare of tissue donors, the need for informed consent and confidentiality, respect for the human body and sensitivity towards the religious and cultural perspectives and traditions of tissue donors. More recently, these principles have been applied by the BAC in a set of more detailed guidance on biobanking and research involving human biological materials [32]. The new HBRA effectively formalises these guidelines, rendering their requirements applicable to all researchers and research institutions operating in Singapore (regardless of the source of funding). The content of these ethical obligations is not substantively different from the broad international framework for ethical research conduct derived from the Nuremburg Code and by the Council for International Organizations of Medical Sciences. What is different is the incorporation of these principles into legislation, giving greater weight in ensuring compliance.

## Taiwan

In April 2005, the Taiwanese government launched a Biomedical Technology Island Plan in which a large-scale population biobank was proposed as a governmental project to support biotech development and medical research in Taiwan [33]. The Taiwan Biobank aims to collect blood, plasma, urine and DNA samples from 200,000 healthy participants aged 30–70 to link with their lifestyle, family history and health information as a prospective cohort study for the development of personalised medicine [34]. In addition to the 200,000 healthy individuals, the biobank has also planned to collaborate with major hospitals in Taiwan to further recruit 100,000 participants from patients over the next decade [35]. The collected data aims to be used to study the 12 most common complex diseases among Taiwanese, including breast, lung, colon, and liver cancers, stroke, Alzheimer and chronic kidney diseases. The Taiwan Biobank is funded by the Ministry of Health and Welfare (MOHW) and was established at the Institute of Biomedical Sciences (IBMS) at Academia Sinica - the highest national research institute in Taiwan.

In 2010, the Legislative Yuan (Parliament) passed the *Human Biobank Management Act* (HBMA) as a legal framework to regulate the establishment, management, and applications of all types of biobanks in Taiwan. The Act stipulates rules on informed consent and data security, and requires that a biobank operator shall establish an ethics committee to review and supervise matters related to the management of biobanks, including applications for access to data and information stored in the biobanks [36]. In addition, it requires an external approval from an expert review board, which is organised by the competent authority, to supplement the internal review mechanism. The Act also stipulates rules on benefit sharing for any profits derived from commercial use received by an Operator and Biobank to the specific population groups [36]. Finally, the Act includes penalties for breaches of rules in the HBMA, specifically, if a biobank operator breaches confidentiality by disclosing participant identifiable personal data or information obtained as a result of the research. Penalties will also be imposed if a biobank operator fails to regularly publish biobank research results or fails to establish an ethics committee and submit prescribed biobank matters to the ethics committee for review and supervision.

## United Kingdom

Continuing its commitment to public investment, the UK Government announced in the 2015 Spending Review that it will invest over £5 billion in health research and development by 2020 [37]. The Medical Research Council (MRC) is a key funder in a variety of partnerships including biobanking [38], and in addition to the government, the Wellcome Trust (WT) is the UK’s largest non-governmental source of funds for biomedical research, with donations totalling approximately £13.9 billion [37]. In 2014, a consortium of funders under the leadership of the MRC established the National Tissue Directory and Co-ordination Centre, with the aim of establishing a directory of biobanks in the UK [39].

Following the success of the Human Genome Project in the late 1990s, the UK government has invested heavily in biobanking to translate clinical outcomes from genomic data. New genetics research partnerships between the government-funded National Health Service (NHS) and industry have been proposed since The NHS Plan [40] and following considerable investment in systems to collect standardised and comparable data on clinical history, consultations and investigations, and to allow linkage across different data sets [39]. The existence of the NHS has meant that the UK is uniquely well-positioned to generate valuable epidemiological data [39].

The UK government (Department of Health), the MRC, and the WT, together with the British Heart Foundation, the Scottish Government, the Welsh Assembly and the National Regional Development Agency have invested £90,711,541 to date in UK Biobank: the world’s largest large-scale, publicly funded, population biobank. Core funding (MRC and WT) has been extended from July 2015 to the end of 2017 [41]. UK Biobank supports investigation into a range of common diseases occurring in the UK. Half a million participants have given broad consent to the use of data collected at recruitment, and can re-consent for supplementary data collections (e.g., brain imaging study). Nearly 100,000 samples have been genotyped for research use as part of the project [42].

The UK10K Rare Genetic Variants in Health and Disease is a project established to use existing research samples to characterise the genetic bases of rare diseases through comparison of genotypes of affected individuals with deeply phenotyped groups from cohort studies [43]. The National Cancer Research Institute (NCRI) Confederation of Cancer Biobanks is a consortium of biobanks and bio sample collections based in the UK [44].

Despite the UK’s leading position in biobanking, there is as yet no specific legal framework for the governance of genomic databases/biobanks. Instead, there is guidance from the Health Research Authority that apply to biobanks which mean that research ethics approval is not needed for the use the samples stored in the biobank as the biobanks take on responsibility for oversight of the use of tissue as part of their Human Tissue authority licence [45]. In the UK, there exists a multi-dimensional nexus of law that applies to medical research on human beings, based on a distinction between human material (samples) and information relating to individuals and their data [46].

## United States of America

In the USA, biobanks have emerged as a key feature of the research landscape over the past 15 years. In the most comprehensive assessment to date in that country, Henderson and colleagues surveyed 456 biobanks (out of over 630 recruited participants) and observed significant heterogeneity in organisational structure, size, purpose and financial models. While most biobanks are affiliated with academic and medical research institutions, biobanks tied to public health delivery systems (Kaiser, Mayo, Geisinger, Veteran’s Administration etc.) and private entities, and numerous smaller scale and disease-specific biobanks have also proliferated. The National Institutes of Health (NIH) continues to be the premiere source of funding and support for biobanks across the country and the key locus of major biobanking efforts such as those based at the National Cancer Institute [47].

The fractured system of health care delivery and payment in the USA presents an impediment to creating a population biobank akin to countries with more centralised systems (e.g. UK, Canada, Estonia). However, the NIH’s recently announced Precision Medicine Initiative Cohort Program envisions a million individual strong longitudinal cohort of research participants who will be recruited, in part, by networking some of the aforementioned biobanks with newly funded collections [48]. Additionally, government public health departments (particularly in the past 10 years) have developed biobanks comprised of residual samples from public health surveillance programs such as Newborn Screening programs. These DBS biobanks generated controversy in two states (Texas and Minnesota) resulting in the unfortunate destruction of several million samples and the concomitant failure to build public support for this research endeavour [49]. Still the interest in turning this resource in to a “research goldmine” [46] remains high as several states continue to explore developing such biobanks [50, 51]. Further the Newborn Screening Translational Research Network was created at the NIH in order to help the States that have created biobanks for the use of residual samples to connect with researchers interested in using them.

In 2015, amendments to the federal guidelines that govern human subjects’ research protections (known as the *Common Rule*) were proposed and are subject to public comments. The controversial revisions, among other measures, would require broad consent for all secondary research utilising biospecimens. This change would apply even when the samples are de-identified, thereby eliminating one of the traditional exemptions for population health studies [49]. The impacts of this change to the Common Rule for biobanks are not yet known, though the prospect of gathering broad consent for use of samples received mixed reviews from contributors to the government’s open call for comment on the proposed changes.

## The first wave – establishing biobank governance and management frameworks

The initiatives of the countries, described above can be seen as part of wider international trends and governance responses to biobanking and the development of research infrastructure. In each of these countries there has been considerable investment in building biobanking co-ordinating networks as well as national and regional biobanks. These biobank collections represented a culmination of bioinformatic and biotechnological advancements that have enabled storage of samples and data, and linkage of data on a hitherto unprecedented scale. This, in turn, entailed a shift from individual research projects to biobanks as platforms to support longitudinal research projects. The typically large-scale and ongoing nature of these complex biobanks also heralded concerns in relation to consent, privacy, and other governance and security issues. Biobanks also enlivened debates about ideas of public and private good [52, 54, 55]. Whilst the establishment of population biobanks, as resources for a vastly increased number of researchers, is well recognised as one of their key public interest functions, their establishment also led to the development of bespoke governance models that have addressed the particular legal and ethical issues that they raise.

The emergence of biobanks for longitudinal research created a major challenge to traditional ideas of consent in health research [53, 56, 57]. Approaches to obtaining consent from participants had to be reconceptualised; primarily because, at the time samples are collected, future research uses are unknown. The very nature of biobanks as research resources means that they will involve prospective as yet unspecified research projects. From a practical point of view, obtaining specific consent from each participant for every separate research project for which his or her samples and information are to be used is challenging. This has led to a growing acceptance of the notion of broad consent to future unspecified research, as a practical solution with specific consent for the taking of a sample and information and its storage [51, 56]. However, it will be shown later in this paper that new, more nuanced approaches to consent are emerging.

Other debates included how to guard against potential re-identifiability through sample coding [58]. The use of linked data is a key element of the usefulness of biobanks, but at the same time this presents risks to participants’ confidentiality and privacy, particularly taking account of the special and sensitive nature of human genetic information [59]. Privacy law was and remains a principal regulatory framework for biobanks, based on the influential Organisation for Economic and Cultural Development (OECD) *Information Privacy Principles*, published in 1980. Broad adoption of these OECD principles brought a measure of consistency to national approaches to privacy.

Apart from the privacy regime, national ethical codes for research conduct generally underpinned a comprehensive national regulatory framework for the ethical conduct for research covering all activities for the collection, storage and distribution of human tissue samples and data with guidelines on consent to the use of stored data for research. Public trust also emerged as an imperative for biobanks [60, 61]. It became clear that establishing and retaining public trust was central to the success of biobanks, especially in the case of population biobanks. This was demonstrated in the failure of one of the earliest population biobank, the Icelandic Health Sector Database (HSD) after the Iceland Supreme Court ruling [62].

At this time, an effective governance framework became widely accepted as an essential prerequisite to engendering and maintaining public trust. Early in the ‘biobank revolution’, policy makers and commentators began to recognise that the biobank phenomenon stretched general research ethics guidelines, and that specific governance frameworks were needed to guide policy development across a range of issues including consent, privacy and access. These, in time, have emerged. Internationally, the OECD *Guidelines on Human Biobanks and Genetic Research Databases* were published in 2009 [63] and were contemporaneous with an expansion of international collaborations. However, the variability of biobank collections (e.g. size, scale, health status of participants, scope of potential research and nature of the collection) has presented challenges for consistent regulatory responses.

These early biobanking initiatives and governance frameworks were also influenced by local experience, determined by local history, healthcare arrangements, funding and other factors. It was recognised nationally that improved governance practices were needed in the form of external regulatory layers from legislation, ethics codes and other codes of practice and guidelines, as well as internal institutional governance arrangements. Consistent with the OECD best practices [63], biobank governance arrangements generally include an oversight body and a system of regular reviews to ensure compliance with developing governance, ethical and legal standards [64].

## The second wave - collaboration and standardisation

The good governance wave in biobanking was paralleled by an increased professionalisation of biobanking as an ‘emerging scientific and operational area’ in research [65] and the development of quality management and standards in sample collection, processing, storage and data management. By the first decade of the 21st century, it was recognised that biobanks needed to develop more standardised and harmonised technical procedures [66]. Biobanks provide platforms for collaboration [67]. Collaborative networks within the biobanking landscape are widely seen as the most cost effective means of accelerating translational research. Considerable investment was made in projects that encouraged standardisation and co-ordination of activities at national, regional and international levels.

## International and national collaborative research networks

In Europe, two key collaborative mechanisms were the Promoting Harmonisation of Epidemiological Biobanks in Europe (PHOEBE) [68] and the Biomolecular Resources Research Infrastructure (BBMRI) initiatives. PHOEBE was a collaboration promoting harmonisation of epidemiological biobanks, which ended in 2009. The BBMRI was established as an EU biobank infrastructure project building on existing resources and technologies. The main aim of BBMRI was to develop an information technology concept for the exchange of data between biobanks (at national and European levels) and strategies for biobank material quality management, and also to present a positive and transparent image of biobanking. The BBMRI is now recognised as a European Research Infrastructure Consortium (ERIC), pulling together a broad range of biobanks operating to increase efficacy and excellence in research of European interest [69]. A number of European member states are full members of ERIC including the UK that joined recently in 2015 [70].

At a national level, there are a number of examples of co-ordinating activities. In Germany biobank operators have been maintaining and updating their data in an interactive manner in the German Biobank Registry (GBR) which is a member of BBMRI-ERIC [71]. In the USA, the Coriell Personalized Medicine Collaborative (CPMC) [72] aims for better understanding the impact of genome-informed medicine by combining a biobank facility with modern microarray technology. Similarly, the Australasian Biospecimen Network Association (ABNA) established in 2001, provides online support for those managing and engaged in biobanking to share information and organises an annual conference on current topics. In Taiwan, a similar effort in networking can be found in the Taiwan Clinical Trials Consortium (TCTC) that was set up by the National Research Programme for Biopharmaceuticals (NRPB) for promoting clinical data sharing and integration for pharmaceutical applications. In Singapore, the Singapore Tissue Network (STN) was established in 2002 but was discontinued in 2011, whereas in the UK, a co-ordination network has just been established [39].

## Standardisation

It became apparent that in order to derive optimal value from these collaborative initiatives as a collective resource, standardisation of policies, practices and procedures would also be required. This lead to the creation of the Public Population Project in Genomics (P3G) [73], an initiative funded by Genome Canada [74] to facilitate collaboration between many national biobanks and to provide a public and accessible knowledge database for the international population genomics community.

At the international level, the International Society of Biological Environmental Repositories (ISBER) has taken a leading international role in standardising preservation and storage of biobanked material. The ICGC is an exemplar of efforts to facilitate and integrate data exchange for close to 200 large-scale cancer research projects. ICGC developed a regularly reviewed set of guidelines for both open and controlled-access data sets. Additionally, the World Medical Association (WMA) is in the process of preparing a *Declaration on Ethical Considerations regarding Health Databases and Biobanks*.

In should be noted, however, that standardisation processes sometimes face strong resistance, particularly from smaller biobanks because of concerns that they might not be able to meet the benchmarks, resulting in a threat to their existence. However, the trend in latest funding guidelines shows that the establishment of comprehensive and generic standards for the relevant fields of biobanking is required, including information technology networking, quality management, responsibilities towards the public, advising biobanks, education and training and last, but not least, considering ethical, legal and social issues, such as the German system [75].

The standardisation of responses to significant ethical, legal and social concerns is increasingly being seen at the global level. UNESCO's International Bioethics Committee (IBC) for example, considered, in its session 2013, the need to update its previous reflections on the human genome and human rights and to put a special focus on biobanks during its future work [76]. In its latest report of September 2015, the IBC provides a brief description of the ethical challenges associated with biobanking and provides practical recommendations for an international registry of all existing biobanks, the conditions of the moral acceptability of a broad consent from biobank participants and the specific points for a model of governance for biobanks. It calls on states and governments to develop a trustworthy form of governance for biobanks and a biobank secrecy but also to harmonise the corresponding rules on data confidentiality and ethics review at the international level [77].

## The third wave – seeking sustainability amidst on going challenges

At the early biobank establishment stage, and amidst initial enthusiasm, issues regarding long-term viability, and strategies for discontinuance of biobanks were not at the forefront, unlike today, when policies emphasise the importance of addressing such issues from the outset. Indeed, over the years, there has been increasing focus on the responsibilities of biobanks with regard to requirements for establishment, communication with participants and returning results back to them. It has required a very delicate balance to get the settings right for biobanks in order to promote the full gamut of genomic research opportunities that they are capable of supporting, and at the same time ensuring adequate governance arrangements. Despite this progress, many of the practical questions as well as ELSI raised in the first wave of biobanking continue to be debated. There are also contested new debates regarding return of individually relevant results [78–80] and incidental findings [81–84].

## Sustainability concerns

At this time, it became evident that biobanks needed to be more focussed on developing and maintaining sustainable business practices. In an environment of rapid technological change this has proven a demanding task. Over a decade ago, Professor Hank Greely observed that biobanks could be “staggeringly expensive” [85]. For example, the National Cancer Institute has been said to spend over $50 million a year on its basic biospecimen resource infrastructure [86]. Vaught and colleagues have insightfully noted, the “[t]ight economic realities in clinical and research operations have spurred the need to re-examine financial models that support the infrastructure of biobanking” [7].

In the Australian context, despite initial enthusiastic adoption of biobanking, this country is undergoing a “levelling off phase” where biobank sustainability and continued funding are certainly emerging as key national issues. The NHMRC established a National Biobanking Strategy Committee that met during 2012–2013 and recognised that, without a more stable core-funding stream, the viability of many biobanks, especially those established for cancer research, could not continue. In Germany, the German Biobank Symposium, co-organised yearly by the GBN since 2012 and established as the specific national experts’ event for biobank research, put a focus in 2013, inter alia, on the question of business and financial models for biobanking. It noted that even well established, financially well-positioned biobanks need long-term sustainability concepts to be able to maintain and provide samples [87]. Long-term sustainability calls for setting up biobanks as research and technology platforms in an interdisciplinary manner across different research centres [88]. In Singapore, the SBB was discontinued in September 2011 as this large-scale research infrastructure was evaluated to be unsustainable operationally and financially, due to a number of concerns including under-utilisation [29].

Sustainability is not a universal challenge and some biobanks are clearly surviving and prospering. The UK Biobank has had its funding extended [41] and, in Taiwan, the Ministry of Health and Welfare (MOHW), the main funding body of the Taiwan Biobank has secured the funding for the next decade, demonstrating the state’s plan to use biotechnology as its developmental niche. The new funding agency of Japan, Agency for Medical Research and Development (AMED), also continue to support three major biobank projects with 5.1 billion yen for FY2016 though the funding is only for maintaining the core biobank activities. Similarly, the Biomolecular Resources Research Infrastructure (BBMRI) was not only recognised as a European Research Infrastructure Consortium but also awarded European legal status in December, 2013 [69] Nevertheless, many biobanks were subject to an “underlying belief that at some point, [they] should be capable of becoming ‘self-sustaining’”[1] but this model is not often achieved.

Cost-recovery has been touted as obvious solution to this issue. Cost-recovery usually means a minimal fee is charged, particularly for academic researchers, whist an often significantly higher fee is charged of researchers from commercial entities [e.g., [89]]. However, the few reports on cost-recovery are not encouraging. The Australian Breast Cancer Tissue Bank, for example, has a cost recovery policy and process policy but recovers negligible amounts of fees in relation to its operational budget. Similar gloomy reports have been made by Canadian biorepositories [90]. There is further concern relating to cost-recovery; that many biobanks are not fully being used for the purposes for which they were established. In particular, the smaller biobanks tend to have few requests for access [91]. This impacts on any success that a cost-recovery model might have. Arguably, a measure of biobanking success is the number of outgoing samples [92]. Methods that have been suggested as means to overcome this under-use problem include, providing a “catalogue” of samples [91] and increased importance of advertising and “market research data” [1].

## Public trust and commercialisation

A business model with a more commercial focus might address some of the biobank funding sustainability issues, however, such models may come with potential grave consequences, if not introduced with caution as in some countries commercialisation is prohibited. Past research would suggest that the shift to commercialisation of medical research is accompanied by unique, often problematic, concerns [60, 93, 94]. Loss of public trust is a particular concern whenever commercial considerations enter the equation [95, 96]. The difficulty of balancing commercialisation and biobanking is aptly summarized in by Turner and colleagues: “Biobanks are caught directly between the values and rights of the participants and the potential commercial and scientific value of the samples and data, and, at the same time, have to construct a business model that will ensure the long-term sustainability of the biobank” [97].

Redefining commercialisation activities and focussing on the creation of knowledge rather than commodities realigns commercialisation with notions of public good. Qualitative research using deliberative democracy methodology has shown that it is possible to counter the ‘natural prejudice’ that many people have against commercialization through independent governance of biobank resources and transparency with regard to commercial involvement [98]. This is notwithstanding that public trust in biobanks is inevitably reduced where there is a commercial partner, particularly an international commercial partner [94]. Indeed, loss of public trust has been connected with the demise of certain biobanks in the past [67].

## A need for new business models?

Integrating successful business strategies into biobanking practices may change the practice of biobanking. Use of advertising and marketing metrics have been suggested as means to attract new funding partners. Commercial marketing strategies may also have the added benefit of re-invigorating traditional research sponsors. Consequences of this integration are likely to shift the focus of biobanking to fulfilling the metrics that result in funding success. This may have unintended consequences if metrics are not reflective of the activities which provide public benefit, or better health; the reason for which they have been created. For example, if the metrics are heavily skewed towards profit making activity it may influence the types of practices undertaken including preferring to sponsor industry-driven research above entirely academic pursuits. Implementing models that appear to commercialise or commodify biobanking (and the samples they contain) may also negatively affect public perceptions and donor support and erode public trust. If metrics that measure biobank success are to be introduced, they should be carefully considered so that they may be accurately measured, while at the same time reflect actual societal value in order to be effective and useful long term. Further, should there be more commercial connection in biobanking more research is required as to how to maintain public trust.

Watson and colleagues have suggested that there are a number of metrics that could be used to assist in measuring the sustainability of biobanks. These may include “financial value”, “operational efficiency” and “social acceptability” [1]. They argue for better differentiation of the categories of biobanks, so that the metrics are particular to the type of biobank (i.e. user, size, kind). They also tentatively suggest that metrics for measuring biobank sustainability should include value to society [1]. One suitable metric for measuring public benefit could be success in research discovery. Another measure could be actual use, or number of requests to access biobank resources a biobank. Further metrics need to be developed to address the competing, but ultimately compatible, interests of funders, researchers, participants and other stakeholders, as well as the wider community, to put a measure on biobank “value” [7].

Standardisation and accreditation may assist in enhancing the value of biobank resources and improving their long-term sustainability. For example, many have argued that standardisation or accreditation of human tissue sample collections would improve the “quality” of results, and would likely lead to increased applications for use of biobank resources operating under these conditions [99]. Accreditation schemes of this nature are being debated by international groups, such as ISBER [95]. To date, there is no consistent standardised biobank model which is generally accepted nor which is proven to be the optimal paradigm. Whilst it is unlikely that there will be a single business or operational biobank model, greater consistency is clearly a desirable goal.

Underlying this “business model” analysis is a supplementary but fundamental question on whether human tissue banks are still necessary. The usefulness of physical human tissue samples for research needs to be considered against the backdrop of advancing technology in whole genome sequencing (WGS) and its increasing accessibility. There is much promise in the information provided by WGS. Some even argue that WGS data potentially removes the need to keep physical tissue. However, the case for retention of physical human tissue remains compelling and persuasive. Firstly, without standardisation across samples, it is important that researchers have access to material to ensure consistent methods across materials in a study. Secondly, the reliability of single WGS scans still depends upon the reliability of the system and platform that produces the results. Thirdly, modern genomic researchers are now accepting the critical importance of linking genomic data to clinical and environmental data. The WGS scans require supplementation and accurate links to relevant lifestyle and other information. However, if convincing evidence emerges showing that access to tissue is no longer necessary this would dramatically impact on the need for biobanks and would shift the focus away from storage of biospecimen to data banks.

## A fourth wave – biobank futures and new biobank models

There are a number of recurring themes in biobank debates that must be taken into account in considering the future of biobanking. These include, but are not limited to: ongoing problems with the nature of consent particularly whether broad consent is ethically defensible; ensuring respectful and appropriate ongoing involvement and connection with participants; retaining public trust in an increasingly commercialised research environment; properly maintaining the physical space required to store tissue; keeping up to date with rapidly changing technology that may result in more accurate collection/storage/analysis; and, perhaps most problematically, sustainability in a constrained funding environment [100].

The recurring debates regarding informed consent have marshalled the concept of “dynamic consent”. Dynamic consent utilises modern communication techniques to facilitate the recognised need for ongoing interaction between a researcher and participant, allowing them to make specific decisions regarding types of research and participation (or withdrawal). According to Kaye and colleagues,

Dynamic consent is a personalised, communication interface to enable greater participant engagement in clinical and research activities…This approach is ‘dynamic’ because it allows interactions over time; it enables participants to consent to new projects or to alter their consent choices in real time as their circumstances change and to have confidence that these changed choices will take effect [96].

One benefit of building dynamic consent into the biobank model would be the capacity of greater interactions with participant. Arguably, participants benefit because their choices are respected over their lifetime and their contributions to the biobank can continue to be utilised for research purposes. Researchers may also benefit from using this model for consent in a number of ways. First, dynamic consent and related approaches to ongoing contact may allow researchers to monitor the health of the participant and their family members over time. Secondly, where limitations in original consent documents may have otherwise prevented researchers from undertaking new types of research, participants could be recontacted for dynamic re-consent. It may also facilitate recontacting of participants and their families to acquire new tissue samples. This would be especially important in the context of cancer tumours where each type of cancer is increasingly understood to have its own distinctive heterogeneous genome identity. It is increasingly important that tumours are available for sequencing. Whilst the dynamic consent model clearly holds much promise, its benefits require a cultural change for researchers and investment in software that could be offset by reduced costs of recruitment, re-contacting and re-consenting.

Building on the dynamic consent model, we propose the concept of a ‘walking biobank’. The basis of this idea rests in substituting the collection and long-term storage of tissue for the ongoing engagement of the participant in genomic research. On this model the research participants themselves serve as the storage units of their genomic material and the researcher, rather than expending limited funds on the infrastructure to maintain specimens in suspended animation, invites participants in a contact database (through dynamic consent models of contact) to ‘walk in’ to donate tissue or information as required to address a specific research question. The feasibility of the ‘walking biobank’ model, of course, rests on continuing reductions in the costs of DNA sequencing such that it will be cheaper to sample, generate genomic information, store the resulting data ‘in silico’, and discard the leftover tissue, than to store that tissue for (only potential) additional analysis. Sample collection and use would, therefore more closely resemble the way that blood samples obtained for clinical purposes are currently analysed and discarded once results are verified. This ‘walking biobank’ model is not without limitations. First, a key benefit of the traditional model is that once participants have provided their sample they need have no further involvement with the biobank, should they so choose. Nevertheless, if sample collection were straightforward the model would have the benefit of reminding participants of their continuing involvement in research, which may be overlooked/forgotten on the traditional model. Secondly, the walking biobank model relies on participant willingness to donate ‘on demand’, so placing a significant burden on them, depending on the nature and frequency of requests made by the biobank. However, with many biobanks struggling to identify users interested in accessing currently stored samples, this may or may not turn out to be a salient concern. Certainly, returning repeatedly to the same participants for re-donation would increase the need for biobanks to prove to participants that they should remain active, and to keep their trust and engagement at a high level. Thirdly, there are related sustainability, cost and efficiency arguments with such on-going checks and additional sample collection. Participant recontact, attendance at the biobank and donation would involve additional costs. Finally, the ‘walking biobank’ model suggests that the death of a participant would prevent further biospecimen collection and possibly see the demise of the biobank that spans generations (although the maintenance of derived data in databases would permit continuing cross-generation comparison).

Some biobanks have already adopted a type of ‘hybrid model’ involving both maintaining tissue collections and long term relationships with participants and their families. For example, kConFab is a large breast and ovarian cancer repository based in Australia, where. The repository staff has formed close connections with donating families who regularly provide additional tissue and health or lifestyle information. Effectively, such repositories include aspects of the ‘walking biobank’ into their operational model but have yet to move to more dynamic consent and still rely heavily on broad consent, facilitated through strong trust between participants and the repository.

Another biobank initiative, referred to as the ‘virtual biobank’, involves creating better indexes and data catalogues. Such improved data management systems have led to the emergence of another new model. This has been described as an ‘electronic database of biological specimens and other related information, regardless of where the actual specimens are stored’ [100]. The ‘virtual biobank’ model has the potential for broader use of biobank resources by better connecting researchers to the required materials, thus minimising duplication. However, this ‘model’ does not resolve the significant cost issues in relation to maintaining physical banks of tissue, as it is based on the connection between, and sharing biobank resources. The ‘virtual biobank’ model also highlights the clear need for consistency and accreditation of biobanks. There are some examples of this virtual model in the USA, where the Office of Biorepositories and Biospecimen Research (OBBR) created the Biospecimens Research Network (BRN) and a Biospecimen Resource Database (BRD). Once populated, it will operate as a virtual biobank [97], but the funding for the various components of the BRD is a matter of ongoing uncertainty. However, if numbers of applications increase significantly, this may reinvigorate attempts to introduce a cost-recovery model. In addition, in Sweden the BARCdb alongside a database of samples, plans to offer guidance in standardising state of the art processes to increase value and utility in samples [98]. This, or similar standardisation, may provide sufficient basis for a virtual model to exist and promote increased usage.

## Conclusion

This article does not propose that the biobank bubble has burst, but it suggests that there are challenges that require mechanisms to be put in place to transform current practices. It is predicted that the vastly expensive population-wide biobank model is unlikely to survive in its current form for much longer. Much academic literature details potential solutions, some outlined in this article, for the ongoing challenges experienced by biobanks. Biobanks have attracted considerable investment over the past twenty years, as they have been perceived as having the potential to accelerate research and reduce the time and costs of research by providing ready access to large numbers of samples and data. More recently this has been questioned. Biobanks have not reached a stage of long-term sustainability even though it had been thought initially that the burden of sustainability would ease over time. This does not appear to be the case, although reliable assessment is difficult to attain. Many of the international and national bodies assisting biobanks to solve emergent challenges do not have access to the resources required, so individual biobanks are largely left to deal with significant challenges on their own. A commitment by governments and funders to funding biobanking as national, regional and international research infrastructure, from the public purse would offer sustainability. However, this is simply not possible for many countries.

In this fourth wave of innovation and activity, a shift towards a more “customised” approach to biobanking can be witnessed, with national and regional variations depending upon circumstances and political support. There is not yet a single dominant or accepted biobank model but there is potential for aspects of traditional models to be used in conjunction with other new initiatives to build innovative sustainability and business systems responsive to particular biobank needs. Moving to more virtual models and the use of online technologies to support dynamic consent and walking biobanks are ways in which sustainability may be realised.

The evidence tends to support that the way forward will be more individualised responses in terms of sustainability and business models that incorporate quality and operational standards to encourage sharing and facilitate linking, both in the electronic sense and as to the use of physical materials. In this article, successive waves of innovation in biobanking practice have been mapped to provide some possibilities to address sustainability issues in biobanking. The future may be unclear, but what is certain is that sustainability is an issue that needs to be resolved to enable biobanking to continue.

## Abbreviations

A*STAR, Agency for Science, Technology and Research; ABNA, Australasian Biospecimen Network Association; AMED, Agency for Medical Research and Development; AOCS, Australian Ovarian Cancer Study; BAC, Bioethics Advisory Committee; BARC / BARCbd, The Biobanking Analysis Resource Catalogue; BBMRI - ERIC, Biomolecular Resources Research Infrastructure European Research Infrastructure Consortium; BBMRI, Biomolecular Resources Research Infrastructure; BMBF, German Federal Ministry for Education and Research; BRD, Biospecimen Resource Database; BRN, Biospecimens Research Network; cBMBs, five centralised biobanks; chub, Cancer Human Biobank; CPMC, Coriell Personalized Medicine Collaborative; DIISR, Department of Industry, Innovation, Science and Research; DZG, German Centres of Health Research; EDRN, Early Detection Research Network; GBN, German Biobank Node; GBR, German Biobank Registry; Global Alliance, Global Alliance for Genomics and Health; HBMA, *Human Biobank Management Act*; HBRA, *Human Biomedical Research Act;* HSD, Health Sector Database; IBC, International Bioethics Committee; IBMS, Institute of Biomedical Sciences; ICGC, International Cancer Genome Consortium; ISBER, International Society of Biological Environmental Repositories; MOHW, Ministry of Health and Welfare; MRC, Medical Research Council; NBSTRN, Newborn Screening Translational Research Network; NCRI, National Cancer Research Institute; NHGRI, National Human Genome Research Institute; NHMRC, National Health and Medical Research Council; NHS, National Health Service; NIH, National Institutes of Health; NUHS TR, National University Health System Tissue Repository; OBBR, Office of Biorepositories and Biospecimen Research; OECD, Organisation for Economic and Cultural Development; P3G, Public Population Project in Genomics; PHOEBE, Promoting Harmonisation of Epidemiological Biobanks in Europe; SBB, Singapore Bio-bank; STR, SingHealth Tissue Repository; ToMMo, Tohoku Medical Megabank Organization; WGS, whole genome sequencing; WT, Wellcome Trust
